# The impact of perinatal brain injury on retinal nerve fiber layer thickness and optic nerve head parameters of premature children

**DOI:** 10.1007/s00417-023-06069-2

**Published:** 2023-04-29

**Authors:** Yaroslava Wenner, Kira Kunze, Apostolos Lazaridis, Vanessa Brauer, Volker Besgen, Petra Davidova, Walter Sekundo, Rolf F. Maier

**Affiliations:** 1 Department of Ophthalmology, Philipps-University, Universitätsklinikum Giessen and Marburg GmbH, Marburg Campus, Marburg, Germany; 2 Department of Ophthalmology, Goethe-University, Theodor-Stern-Kai 7, 60590 Frankfurt Am Main, Germany; 3 Department of Paediatrics, Philipps-University, Universitätsklinikum Giessen and Marburg GmbH, Marburg Campus, Marburg, Germany

**Keywords:** OCT, Optic nerve, RNFL, Preterm children, Perinatal brain injury

## Abstract

**Purpose:**

This study aims to evaluate the impact of birth weight (BW), gestational age (GA), retinopathy of prematurity (ROP), and perinatal brain injury (PBI) on optic nerve head (ONH) parameters and nerve fiber layer thickness (RNFLT) in preterm children.

**Methods:**

ONH parameters and RNFLT were examined prospectively in 5–15-year-old preterm and full-term children with RTVue-100 OCT (Optovue, USA). The parameters of the two groups were compared and possible influences of BW, GA, ROP, and PBI analyzed in preterm children.

**Results:**

In total, 51 full-term and 55 preterm children were included. The mean age was 9.98 ± 3.4 years in full-term and 10.0 ± 2.5 years in preterm children. The mean GA in preterm children was 29.6 ± 3.8 weeks with a BW of 1523 ± 732 g. RNFLT was significantly lower in preterm than in full-term children in all but temporal quadrants. Cup area, volume, cup/disc area ratio, and horizontal cup/disc ratio (CDR) were significantly larger and rim area significantly thinner in preterm children. GA was positively correlated with superior, nasal, and overall RNFLT and negatively correlated with cup area, volume, and horizontal CDR. ROP stage correlated negatively with superior and nasal RNFLT. PBI was the only significant predicting factor for RNFL thinning in all but temporal quadrant in multiple regression analysis. Preterm children with PBI had a significantly larger optic cup (CDR 0.70 ± 0.33 vs. 0.37 ± 0.27) and thinner optic rim.

**Conclusion:**

PBI correlated strongest with RNFL thinning, a thinner optic rim, and a larger optic cup in preterm children and should be evaluated in each patient to prevent incorrect diagnosis like glaucoma.

## Introduction


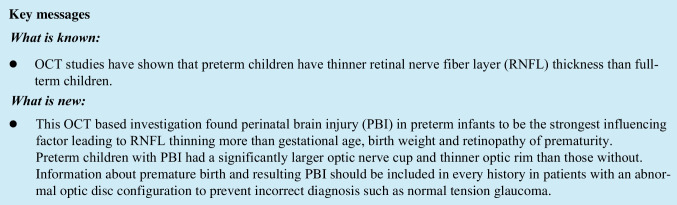


Visual impairment in preterm children is caused mainly by retinopathy of prematurity (ROP) and cerebral lesions [1, 2]. Good monitoring and avoidance of hyperoxia led to a reduction of retinal complications in recent years [3, 4]. Brain injury in preterm infants leads to damage of subcortical white matter, resulting in periventricular leukomalacia (PVL) [5]. Intraventricular hemorrhage (IVH), caused by impaired autoregulation of the cerebral blood flow, is another common cause of neuronal injury in premature infants [5]. Both IVH and PVL can cause retrograde atrophy of the optic nerve, which can result in either a small optic disc or a large optic disc cup, depending on the timing of damage [2, 6–8]. Optic nerve head (ONH) in preterm children often appears pale, with a larger cup and thinning of the neurosensory rim in funduscopic examination [5, 7], which often initiates a suspicion of glaucoma, even in presence of normal eye pressure. This can make parents insecure and sometimes lead to unnecessary topical eye pressure–lowering medication, which can cause local and systemic adverse reactions.

Technical progress allows objective and precise high-resolution in vivo measurements of the peripapillary RNFL and ONH with optic coherence tomography (OCT) in children [9]. Several studies have shown that retinal nerve fiber layer thickness (RNFLT) measured with OCT in preterm children was lower than in full-term children [10–16]. This phenomenon was interpreted as subclinical optic nerve hypoplasia [15]. Besides a hand-held OCT study in very small preterm infants [17], we do not know any other studies that investigated ONH parameters by means of OCT in premature school-age children and their association with central nervous system pathology.

The main goal of this study was the evaluation of the influence of gestational age (GA), birth weight (BW), stage of ROP, bronchopulmonary dysplasia (BPD), and perinatal brain injury (PBI) on RNFLT and ONH parameters. Secondary goal was the comparison of RNFLT and ONH morphology between preterm and full-term children by means of the RTVue 100 OCT (Optovue, Fremont, CA, USA). Better OCT-based knowledge about ONH morphology is very important for preterm born patients since abnormal ONH parameters can be misinterpreted, e.g., as a normal-tension glaucoma in adulthood.

## Materials and methods

### Study design and population

Fifty-five prematurely born children with GA of 36 weeks or less (study group) and 51 randomly selected full-term healthy children with GA ≥ 37 weeks (control group) were included in this study at the age of 5–15 years. Study participants were recruited prospectively mainly from patients presenting on regular basis to the Ophthalmology Department of the University Hospital in Marburg. In addition, hospital records were used to locate prematurely born children treated in the neonatal unit in Marburg, who were invited by letter to participate in the study. Twenty-three children accepted the invitation and agreed to take part in the investigation. Twenty-two of them were included in the study group. In one child, the OCT examination was not possible due to lack of cooperation.

Prior to participation, information was provided to the patients and their parents regarding the nature of the tests and informed consent was obtained. All procedures followed the guidelines of the Declaration of Helsinki and were approved by the Ethics Committee of the Faculty of Medicine, Phillips University of Marburg (No. 85/14).

For the control group, amblyopia, abnormal findings of the anterior or posterior segment of the eye, acute or chronic eye diseases, previous ocular trauma or surgeries, and systemic diseases were defined as exclusion criteria. Inclusion criteria were GA ≥ 37 weeks and a visual acuity ≥ 20/20 (logMAR ≤ 0.0). For the study group, the only exclusion criterion was the impossibility of optic nerve evaluation with OCT due to child’s insufficient cooperation. Information regarding GA, BW, stage of ROP, BPD, and PBI was obtained from medical records.

The children underwent a routine ophthalmological examination including cycloplegic refraction. To assess ONH parameters and peripapillary RNFLT, RTVue OCT 100 (Optovue, Fremont, CA, USA) examination was performed and axial length (AL) was obtained with IOL-Master 500 (Carl Zeiss Meditec AG, Jena, Germany). ONH parameters and peripapillary RNFLT were compared between the control and study group. Furthermore, correlation coefficients between BW, GA, ROP, BPD, PBI, and ONH parameters respectively peripapillary RNFLT were calculated in preterms.

### Ophthalmological examination

The best-corrected decimal visual acuity was assessed at 5-m distance using a Landolt ring chart. Assessment of ocular motility and alignment, cycloplegic refraction, and anterior and posterior segment examination were performed. To achieve cycloplegia, 0.5% cyclopentolate solution was administered three times at 10-min intervals. Retinoscopy was performed 45 min after the first instillation of cyclopentolate drops and the spherical equivalent (SE) was calculated. The AL was calculated using the average of five measurements taken with the IOL-Master 500.

### OCT acquisition

After pupil dilatation, RNFL and ONH analysis was performed using RTVue 100 OCT in a darkened room using an internal fixation target. RTVue 100 OCT uses a superluminescent diode laser with a wavelength of 840 nm. Its axial resolution is 5 μm and its transverse resolution is 15 μm. RNFL was measured along a circle centered on the ONH with a diameter of 3.45 mm and a density of 1024 A-scans. Centering of the optic disc was verified by observing the ONH on the video screen during measurement. The data were processed using the RTVue software and an overall RNFLT and thickness of the superior, temporal, inferior, and nasal quadrant were obtained.

The nerve head map 4-mm-diameter (NHM4) RTVue protocol was used to assess ONH parameters. The three-dimensional disc protocol is a 4 × 4-mm raster scan centered on the optic disc and consists of 101 B-scans, each consisting of 512 A-scans. The software automatically uses this image to draw the contour line of the disc margin by identifying the retinal pigment epithelium edges. NHM4 protocol includes 12 radial scans with 3.4 mm in length (452 A-scans each) and 6 concentric ring scans ranging from 2.5 to 4.0 mm (587 to 775 A-scans each), all centered around the optic disc contour line. With this data, ONH parameters are generated. They include optic disc area, cup area, rim area, cup-to-disc area ratio, cup-to-disc horizontal ratio, cup-to-disc vertical ratio, disc volume, cup volume, and cup-to-disc volume ratio.

One examination was performed on each eye, unless the quality was inadequate and therefore repeated. Only images with adequate centering, sufficient signal strength (> 30), continuous scan pattern with no missing or blank areas, and correct RNFL segmentation were included. Only one eye, primary right or with the best examination quality, was used for analysis. Patients with low-quality OCT recording in both eyes were excluded.

### Statistical analysis

Statistical analysis was performed with SPSS 21 for Windows (SPSS Inc., Chicago, IL, USA) and BiAS (Epsilon 2019, Germany). Continuous variables were analyzed using descriptive statistics. The *χ*^2^ test was used to analyze the association between categorial variables. Normal distribution was tested with Kolmogorov–Smirnov test. In case of normal distribution, *t* test was used to compare independent continuous parameters between the groups; otherwise, Mann–Whitney *U* test was used. Univariate and stepwise multivariable linear regression analyses were used to explore factors associated with RNFLT and ONH parameters of preterm children. For the latter, SE, GA, BW, ROP, BPD, and PBI were included. A *p* value ≤ 0.05 was considered to be statistically significant.

## Results

In total, 106 children were included in the study; 55 were prematurely born (study group), 51 were full term (control group). See Table 1 for further characteristics. Both groups did not differ significantly in terms of age (*p* = 0.572). After correction of age in the preterm group, taking into account the GA, there was still no significant difference in age between study and control group (*p* = 0.846). Mean SE was significantly higher (*p* = 0.003) in preterm than in full-term children. Mean AL did not differ significantly between the two groups (*p* = 0.758). Thirteen of the preterm children had suffered PBI, namely, PVL, IVH, or posthemorrhagic hydrocephalus.

**Table 1:** Basic characteristics of control group (term children) and study group (preterm children)

Basic characteristics	Preterm children (*n* = 55)	Term children (*n* = 51)	Difference between groups (*p* value)
Gender male *n* (%)	31 (56)	26 (51)	0.795
Age at OCT imaging (years), mean ± SD	10.00 ± 2.5	9.98 ± 3.4	0.572
Mean visual acuity (logMAR)	0.03	− 0.02	0.550
Spherical equivalent (dpt), mean ± SD	+ 0.43 ± 2.36	+ 1.72 ± 2.23	0.003*
Axial length (mm), mean ± SD	22.6 ± 0.85	22.6 ± 1.14	0.758
Gestational age (weeks), mean ± SD	29.6 ± 3.8	≥ 37.0	
Birth weight (g), mean ± SD	1523 ± 732		
Maximum ROP stage, *n* (%) Stage 1 Stage 2 Stage 3	6 (10.9) 4 (7.3) 3 (5.4)		
ROP treatment, *n* (%)	2 (3.6)		
Bronchopulmonary dysplasia *n* (%)	26 (47.3)		
Perinatal brain injury, *n* (%) defined as PVL IVH Stage I IVH Stage II IVH Stage III IVH IVH with posthemorrhagic hydrocephalus	13 (23.6) 4 (7.3) 9 (16.4) 1 (1.8) 3 (5.5) 5 (9.1) 4 (7.3)		

Significance level: *p* = 0.05; significant results are marked with an asterisk

*SD* standard deviation, *ROP* retinopathy of prematurity, *PVL* periventricular leukomalacia, *IVH* intraventricular hemorrhage

RNFLT was significantly lower in preterm than in full-term children in all but temporal quadrants (Table 2). The cup area, cup volume, cup/disc area ratio, and horizontal cup/disc ratio (CDR) were significantly larger in preterm children than in full-term children. The rim area was significantly smaller in preterm children (Table 3). There was no significant influence of age on RNFLT in full-term and preterm children with and without adjusting for AL. Univariate linear regression analysis showed a significant positive correlation (*p* = 0.0432; *r* = 0.274) of SE with the superior quadrant RNFLT in preterm children. In multiple regression analysis with backward elimination, no significant association was found between SE and RNFLT or ONH parameters in full-term and preterm children.

**Table 2:** Comparison of peripapillary RNFL thickness in preterm and full-term children

RNFLT *(µm)*	Preterm children median (range)	Term children median (range)	*p* value
Average	101 (58–131)	109 (78–156)	< 0.001*
Temporal	79 (44–152)	81 (57–143)	0.091
Superior	125 (85–168)	140.5 (85–195)	0.001*
Nasal	73 (45–108)	81.5 (48–166)	< 0.001*
Inferior	128 (92–198)	139.5 (107–210)	0.002*

Significant results are marked with an asterisk

*RNFLT* retinal nerve fiber layer thickness

**Table 3:** Comparison of optic nerve head parameters in preterm and full-term children

Variables	Preterm children median (range)	Term children median (range)	*p* value
Disc area (mm^2^)	1.87 (0.45–3.44)	2.04 (1.06–3.25)	0.127
Cup area (mm^2^)	0.26 (0–1.80)	0.15 (0–1.53)	0.043*
Rim area (mm^2^)	1.41 (0.15–3.44)	1.63 (0.90–3.02)	0.002*
Rim volume (mm^3^)	0.19 (0.003–1.52)	0.27 (0.05–1.54)	0.018*
Nerve head volume (mm^3^)	0.36 (0.01–2.03)	0.45 (0.16–1.93)	0.011*
Cup volume (mm^3^)	0.02 (0–0.68)	0.005 (0–0.18)	0.042*
Cup/disc area	0.15 (0–0.91)	0.07 (0–0.50)	0.014*
Cup/disc horizontal ratio	0.50 (0–0.91)	0.39 (0–0.77)	0.036*
Cup/disc vertical ratio	0.44 (0–0.99)	0.32 (0–0.83)	0.053

Significant results are marked with an asterisk

### Correlations of RNFLT and ONH parameters with SE, BW, GA, ROP, BPD, and PBI

In preterm children, there was a significant positive correlation of GA with the superior (*p* = 0.011; *r* = 0.368) and nasal (*p* = 0.028; *r* = 0.322) quadrants and average RNFLT (*p* = 0.025; *r* = 0.326) and a negative correlation with cup area (*p* = 0.007, *r* =  − 0.391), cup volume (*p* = 0.018, *r* =  − 0.348), and cup/disc horizontal ratio (*p* = 0.013, *r* =  − 0.363). BW showed significant positive correlation only with RNFLT of nasal quadrant (*p* = 0.039; *r* = 0.302) and significant negative correlation with the cup area of ONH (*p* = 0.0498, *r* =  − 0.291). Only RNFLT of the nasal quadrant (*p* = 0.015, *r* =  − 0.359) correlated significantly negative and the cup/disc area significantly positive (*p* = 0.038, *r* = 0.314) with the severity of BPD. RNFLT of the superior and nasal quadrants (*p* = 0.013, *r* =  − 0.359) correlated significantly negative and the optic disc area correlated significantly positive (*p* = 0.043, *r* = 0.300) with the severity of ROP.

**Fig. 1:**
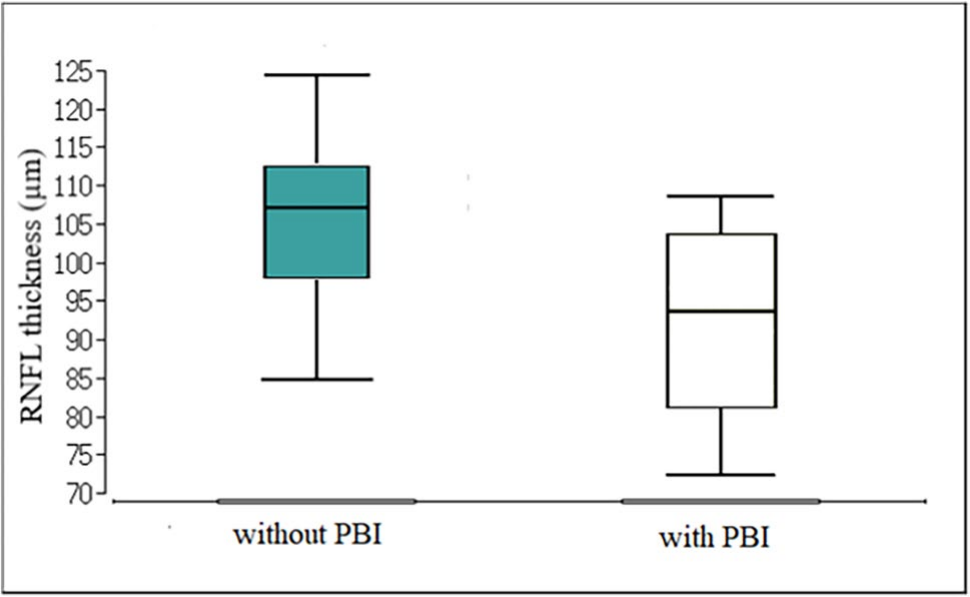
Boxplots of average peripapillary RNFL thickness in 3.4-mm distance from the optic nerve head center in preterm children with and without perinatal brain injury (PBI)

Preterm children with PBI had significantly lower RNFLT in all quadrants as well as average, contrary to preterm children without PBI (Table 4, Fig. 1). In multiple regression analysis with backward elimination, which included SE, BW, GA, BPD, ROP, and PBI, only PBI was a highly significant (*p* ≤ 0.001) predicting factor of RNFLT in all but the temporal quadrant. In ONH parameters, only optic disc area was not significantly influenced by PBI. Preterm children with PBI had significantly larger cup area and volume, cup/disc area ratio, and horizontal and vertical CDR, and significantly thinner rim area, smaller rim, and smaller optic nerve head volume than those without any signs of brain injury (Table 5). In multiple regression analysis, PBI had a significantly (*p* < 0.05) negative correlation with rim area as well as positive correlation with cup area and volume, cup/disc area ratio, and vertical CDR. ROP stage showed significant influence on all ONH parameters except disc area. No significant influence of any other parameter such as SE, BW, GA, or BPD was shown.

**Table 4:** Comparison of peripapillary RNFL thickness in preterm children with and without perinatal brain injury (PBI)

RNFLT *(µm)*	With PBI (*n* = 13) median (range)	Without PBI (*n* = 42) median (range)	*p* value
Average	89 (68–110)	106 (87–131)	0.002*
Temporal	78 (44–86)	82 (61–152)	0.022*
Superior	112 (85–135)	130.5 (95–168)	0.002*
Nasal	64 (45–82)	77 (55–108)	< 0.001*
Inferior	111 (92–153)	137 (98–198)	0.005*

Significant results are marked with an asterisk

*RNFLT* retinal nerve fiber layer thickness

**Table 5:** Comparison of optic head nerve parameters in preterm children with and without perinatal brain injury (PBI)

Optic nerve head parameters	With PBI median (range)	Without PBI median (range)	*p* value
Disc area (mm^2^)	1.83 (1.23–2.56)	1.875 (0.45–3.44)	0.912
Cup area (mm^2^)	0.87 (0–1.63)	0.23 (0–1.07)	0.012*
Rim area (mm^2^)	0.99 (0.15–2.44)	1.49 (0.45–3.44)	0.024*
Rim volume (mm^3^)	0.10 (0.003–0.71)	0.24 (0.038–1.52)	0.011*
Nerve head volume (mm^3^)	0.18 (0.01–1.08)	0.40 (0.09–2.03)	0.011*
Cup volume (mm^3^)	0.13 (0–0.42)	0.01 (0–0.41)	0.010*
Cup/disc area	0.48 (0–0.91)	0.11 (0–0.60)	0.011*
Cup/disc horizontal ratio	0.73 (0–0.99)	0.41 (0–0.92)	0.012*
Cup/disc vertical ratio	0.70 (0–0.98)	0.37 (0–0.91)	0.040*

Significant results are marked with an asterisk

## Discussion

The main goal of this study was to identify the main parameters influencing optic disc structure and RNFLT in prematurely born and to evaluate differences between ONH and RNFLT of preterm and full-term infants.

In contrast to a recently published study of 6–8-year-old Asian children with a sample size of 4034 which found that age was positively correlated with RNFLT [18], there was no significant influence of age on RNFLT in full-term and preterm children in our study. The missing correlation in our study could be due to a smaller sample size in our study.

In our study, preterm children were significantly more myopic than full-term children, but had similar axial length, which was also reported by Lee et al. [12], since myopia in children with ROP is associated with an abnormal development of the anterior segment like steeper corneas and shallower anterior chambers, rather than a long axial length [19]. It is known from literature that the peripapillary RNFLT can be influenced negatively by AL [9, 20] due to a magnification effect in eyes with shorter or longer AL. In this study, we did not perform a correction of the ocular magnification effect, as the AL was comparable and multiple regression analysis did not show significant association between SE and RNFLT or ONH parameters in both groups.

RNFLT was significantly lower in preterm in contrast to full-term children in all but temporal quadrants. This was consistent with results reported in literature [11, 15], which stated that global RNFL was thinner in preterm in contrast to full-term neonates. Similar to our results, temporal RNFLT in the preterm group was 6% thicker in comparison to the full-term group, while all other peripapillary RNFL sectors were significantly thinner in a study by Wang et al. in which 60% of preterm children with ROP were included [15]. This phenomenon might be caused by significant narrowing of the temporal vascular angle of the large retinal vessels emerging from the optic disc in prematurely born children with increasing stage of ROP, which subsequently leads to a different distribution of peripapillary RNFL [21].

BW and, with even higher impact, GA were positively associated with peripapillary average RNFLT in our study. The positive correlation of average RNFLT with BW, as well as with GA, has already been described before [10, 11]. In our study, they were not the main factors influencing RNFLT, as for example stated by Fieß et al. [11]. Our multiple regression analysis showed that the presence of PBI was the strongest influencing factor on RNFLT. In terms of GA, the risk of PBI is inversely proportional to maturity at birth [22], which could, among others, explain the influence of GA on RNFLT.

Retinal ganglion cells start to form the RNFL in the 8th gestational week [23]. Both are supplied by retinal vessels with blood. Thus, a delay of retinal vessel growth can lead to a loss of axons and thus to changes of ONH. It is known from previous studies which analyzed fundus photography that such an early prenatal damage may result in a small optic disc area or larger optic disc cups, depending on the gestational age of brain damage [7]. A further study that analyzed the ONH in fundus photography revealed that optic nerve cups of preterm children with brain lesions were larger in comparison to children without [24]. In our study, preterm children with PBI had significantly larger optic disc cups and a thinner optic rim. The optic disc area was the only ONH parameter that was not influenced by PBI. In multiple regression analysis, only PBI was a significant predicting factor for peripapillary RNFLT. Consistent with our results, in a study using hand-held OCTs in very preterm infants, it was reported that a thinner papillomacular bundle RNFL correlated with higher global brain MRI lesion burden index and lower cognitive and motor scores [16].

To our knowledge, the influence of PBI on ONH parameters measured with OCT has not been investigated systematically before. Due to a lower RNFLT, ONH of preterm children has a significantly smaller rim area and larger optic disc cup. This characteristic is more prominent in subjects with PBI. The knowledge about the strong impact of PBI in preterm infants on ONH morphology, including a thinner RNFL, larger optic nerve cup, and thinner optic rim, can lead to a better interpretation of optic nerve findings in the future. History should include questions about premature birth and resulting perinatal brain injury, especially in patients with an abnormal configuration of the papillae. This could prevent mistaking a large optic nerve cup for, e.g., normal-tension glaucoma in presence of normal intraocular pressure.

### Limitations and recommendations for future studies

The major limitation of our study is the quite small sample size of patients with PBI. More patients would be needed for a more valid comparison between preterm patients with PVL versus IVH. Another limitation is that preterm and full-term children differed at baseline in terms of refraction error which could have been a confounding factor known from previous OCT studies in children [25]. For future studies, the establishment of a normative database of preterm children, especially those with a history of PBI, would be useful, so that clinicians can better interpret OCTs for that population.
